# Supplementary material for: Prognostic Hematologic Biomarkers Following Immune Checkpoint Inhibition in Metastatic Uveal Melanoma
Source: Cancers (Basel). 2022 Nov 24;14(23):5789. doi: 10.3390/cancers14235789 (PMC9738244; doi:10.3390/cancers14235789)
Supplement: Supplementary file 1 [file cancers-14-05789-s001.zip › cancers-1981098-supplementary.pdf]

cancers MDPI

Table S1. Primary Tumor Characteristics, Entire Cohort

Primary Tumor Characteristics			Univariable Analysis			
			OS		PFS	
**Parameter**	**Categories**	**Number (%), *n* = 46 (100%)**	**HR (95%CI)**	***p*-value**	**HR (95%CI)**	***p*-value**
Ocular Location of Primary	Choroid	43 (91.5)	0.19 (0.03, 1.4)	0.10	0.6 (0.2, 1.9)	0.36
	Iris	1 (2.1)				
	Iridociliary	2 (4.3)				
Ciliary Body Involvement	No	23 (50.0)	0.7 (0.4, 1.4)	0.29	0.6 (0.3, 1.1)	0.096
	Yes	21 (45.7)				
	Unknown	2 (4.3)				
Extraocular Extension	No	41 (89.1)				
	Yes	3 (6.5)				
	Unknown	2 (4.3)				
Tumor Thickness (mm)	Median (IQR)	6.7 (5.3)	0.9 (0.8, 1.0)	0.033	0.9 (0.8, 1.0)	0.038
Longest Basal Diameter (mm)	Median (IQR)	16 (2.8)	0.9 (0.8, 1.0)	0.055	0.9 (0.8, 1.0)	0.058
GEP Class	1a (vs 2)	2 (4.3)	3.5 (0.5, 25.9)	0.87	3.1 (0.7, 13.0)	0.64
	1b (vs 2)	2 (4.3)	1.1 (0.2, 5.1)	0.59	1.2 (0.3, 4.7)	0.76
	2 (vs 1)	28 (60.9)	1.3 (0.4, 3.9)	0.63	0.9 (0.3, 2.6)	0.95
	Unknown	14 (30.5)				
PRAME Status	Negative	2 (4.3)				
	Positive	8 (17.4)				
	Unknown	36 (78.3)				
Cytomorphology	Mixed	11 (23.9)				
	Spindle	4 (8.7)				
	Epitheloid	8 (17.4)				
	Unknown	23 (50.0)				
Time from Primary Diagnosis to Metastatic Disease (months)	Median (IQR)	31 (51)				

Table S2. Responders vs Non-Responders, Patient Demographic and Tumor Characteristics

Patient Demographics				
**Parameter**	**Categories**	**Responders , *n* = 10**	**Non-responders, *n* = 34**	***p*-value**
Age (years)	Median (IQR)	59.8 (20.5)	63.9 (20.2)	0.64
Sex	Male	4 (40.0)	20 (58.8)	0.50
	Female	6 (60.0)	14 (41.2)	
ECOG Performance Status	0	10 (100.0)	23 (67.6)	0.046
	1	0 (0.0)	9 (26.6)	
	2	0 (0.0)	1 (2.9)	
	3	0 (0.0)	1 (2.9)	
	4	0 (0.0)	0 (0.0)	
Primary Tumor Characteristics				
Ocular Location of Primary	Choroid	8 (80.0)	33 (97.1)	0.13
	Iris	1 (10.0)	0 (0.0)	
	Iridociliary	1 (10.0)	1 (2.9)	
Ciliary Body Involvement	No	4 (40.0)	18 (52.9)	0.71
	Yes	5 (50.0)	15 (44.2)	
	Unknown	1 (10.0)	1 (2.9)	
Extraocular Extension	No	9 (90.0)	30 (88.3)	1.00
	Yes	0 (0.0)	3 (8.8)	
	Unknown	1 (10.0)	1 (2.9)	
Tumor Thickness (mm)	Median (IQR)	6.4 (6.8)	6.9 (4.5)	0.78
Longest Basal Diameter (mm)	Median (IQR)	17.5 (6.8)	15.9 (2.3)	0.75
GEP Class	1a	1 (10.0)	1 (2.9)	
	1b	0 (0.0)	2 (5.9)	
	2	5 (50.0)	21 (61.8)	
	Unknown	4 (40.0)	10 (29.4)	
PRAME Status	Negative	1 (10.0)	2 (5.9)	
	Positive	0 (0.0)	7 (20.6)	
	Unknown	9 (90.0))	25 (73.5)	
Histopathology	Mixed	2 (20.0)	8 (23.5)	0.56
	Spindle	0 (0.0)	4 (11.8)	
	Epitheloid	1 (10.0)	7 (20.6)	
	Unknown	7 (70.0)	15 (44.1)	
Time from Primary Diagnosis to Metastatic Disease (months)	Median (IQR)	64 (46)	21 (41)	0.32
Sites of Metastasis when ICI therapy was started	Liver	7 (70.0)	32 (94.1)	0.069
	Lung	4 (40.0)	16 (47.1)	0.73
	Brain	2 (20.0)	1 (2.9)	0.13
	Bone	2 (20.0)	8 (23.5)	1.00
	LN	3 (30.0)	10 (29.4)	1.00
	Other	7 (70.0)	12 (35.3)	0.074
	Liver Only	2 (20.0)	12 (35.5)	
	Liver + Extrahepatic. (vs. Liver Only)	6 (60.0)	20 (58.8)	

	Extrahepatic Only.<br>(vs. Liver Only)	2 (20.0)	2 (5.9)

Table S3. Responders vs Non-Responders, Treatment Characteristics

Treatment Characteristics

**Parameter**	**Categories**	**Responders , *n* = 10**	**Non-responders, *n* = 34**	***p*-value**
Enucleation	No	6 (60.0)	15 (44.1)	0.48
	Yes	4 (40.0)	19 (55.9)	
Plaque RT	No	3 (30.0)	16 (47.1)	0.47
	Yes	7 (70.0)	18 (52.9)	
Lines of Prior Therapy	0	10 (100)	31 (91.2)	
	1	0 (0)	2 (5.9)	
	> 1	0 (0)	1 (2.9)	
Immunotherapy	Single ICB	6 (60.0)	27 (79.4)	0.24
	Combination ICB	4 (40.0)	7 (20.6)	0.24
Cycles of ICI Completed	Median (IQR)	11 (7)	5 (8)	0.19
Reason for Discontinuation	Progression/Death	4 (40.0)	25 (73.5)	
	Toxicity	4 (40.0)	7 (20.6)	
	Other	2 (20.0)	2 (5.9)	
IRAE Grade	None	6 (60.0)	16 (47.1)	0.72
	Grade 1	0 (0.0)	7 (20.6)	
	Grade 2	3 (30.0)	6 (17.6)	
	Grade 3	1 (10.0)	5 (14.7)	
	Grade 4	0 (0.0)	0 (0.0)	
Liver Directed Therapy	None	5 (50.0)	16 (47.1)	1.00
	Surgical Resection	0 (0.0)	2 (5.9)	
	SBRT/RT	3 (30.0)	6 (17.6)	
	TACE	1 (10.0)	5 (14.7)	
	IR Y90	0 (0.0)	1 (2.9)	
	Multiple	1 (10.0)	4 (11.8)	
Objective Response Rate	CR	1 (10.0)	0 (0.0)	< 0.001
	PR	1 (10.0)	0 (0.0)	
	SD	8 (80.0)	0 (0.0)	
	PD	0 (0.0)	34 (100.0)	

IRAE, Immune-related adverse event

Table S4. Responders vs Non-Responders, Serologic Biomarkers

Hematologic Biomarkers				
**Parameter**	**Categories**	**Responders , *n* = 10**	**Non-responders, *n* = 34**	***p*-value**
LDH at Stage IV Diagnosis	Median (IQR)	178 (127)	211 (88)	0.14
	WNL (< 240 mg/dL)	7 (70.0)	21 (61.8)	1.00
	Elevated (> 240 mg/dL)	3 (30.0)	10 (29.4)	
	Unknown	0 (0.0)	3 (8.8)	
LDH at Baseline	Median (IQR)	195 (454)	199 (88)	0.6
	WNL (< 240 mg/dL)	7 (70.0)	16 (47.1)	0.25
	Elevated (> 240 mg/dL)	2 (20.0)	16 (47.1)	
	Unknown	1 (10.0)	2 (5.9)	
LDH on Treatment	Median (IQR)	202 (99)	258 (231)	0.24
	WNL (< 240 mg/dL)	7 (70.0)	11 (32.4)	0.041
	Elevated (> 240 mg/dL)	1 (10.0)	14 (41.2)	
	Unknown	2 (20.0)	9 (26.5)	
NLR at Baseline	Median (IQR)	2.6 (7.5)	2.6 (1.3)	0.85
	Below Median	5 (50.0)	15 (44.1)	1.00
	Above Median	5 (50.0)	17 (50.0)	
	Unknown	0 (0.0)	2 (5.9)	
NLR on Treatment	Median (IQR)	2.0 (2.4)	3.3 (2.0)	0.47
	Below Median	5 (50.0)	11 (32.4)	0.71
	Above Median	4 (40.0)	14 (41.2)	
	Unknown	1 (10.0)	9 (26.4)	
dNLR at Baseline	Median (IQR)	1.7 (1.3)	1.7 (0.8)	0.67
dNLR on Treatment	Median (IQR)	1.4 (1.7)	1.9 (1.2)	0.98
**Δ**NLR on Treatment	Median (IQR)	0.3 (2.2)	0.8 (2.0)	0.50
ALC at Baseline	Median (IQR)	2.1 (2.1)	1.6 (0.7)	0.31
ALC on Treatment	Median (IQR)	2.1 (1.9)	1.6 (0.8)	0.57
ANC at Baseline	Median (IQR)	5.6 (3.6)	3.9 (0.9)	0.26
ANC on Treatment	Median (IQR)	5.7 (4.4)	5.1 (3.2)	0.36
AEC at Baseline	Median (IQR)	0.2 (0.3)	0.1 (0.2)	0.71
AEC on Treatment	Median (IQR)	0.2 (0.3)	0.1 (0.2)	0.55

LDH, lactate dehydrogenase; NLR, neutrophil:lymphocyte ratio; ALC, absolute lymphocyte count; ANC, absolute neutrophil count; AEC, absolute eosinophil count

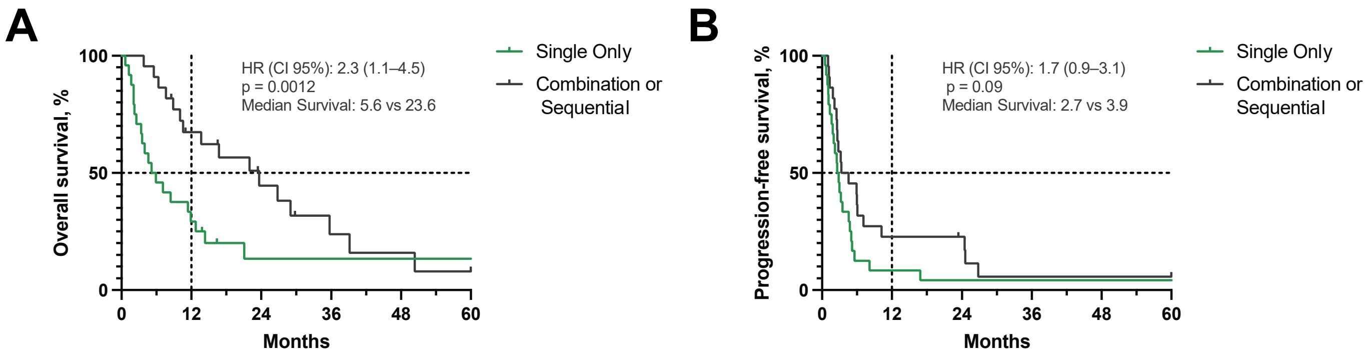

Figure S1. Kaplan-Meier curve of OS and PFS stratified by ICI agent. Single corresponds to lifetime single agent therapy, either Ipilimumab, Nivolumab, or Pembrolizumab. Combination or Sequential corresponds to any patient who received an anti-CTLA4 and an anti-PD1 agent at any point during their treatment course. Dashed lines represent 50%- and 12-month survival points, respectively. Hazard ratios (HR), *p*-values, and median survival are displayed in the upper right corner of each respective graph.
